# A System of Clinical Medicine

**Published:** 1843-04-01

**Authors:** 


### A SYSTEM OF CLINICAL MEDICINE. By *Robert James Graves*, M.D. one of the Physicians to the Meath Hospital, Dublin. 8vo. pp. 938. Dublin and London, Jan. 1843.

THE brief title-page and the ample volume of important matters that follows it, present a remarkable contrast with the generality of publications that come before us, where the crowded first page promises every thing, and the atrophied or tumid body of the work performs nothing! The ample opportunities which Dr. Graves has had in the MEATH and SIR P. DUN'S Hospitals for clinical observation and *experiment*, as well as experience, have not been neglected; and the various detached essays and successive series of lectures which our author has published in the Dublin Hospital Reports, and in the pages of our contemporary, the MEDICAL GAZETTE, shew the diligence, the ability, and the discrimination with which he has gleaned from the bed-side of sickness, and from midnight meditation, a vast mass of the most valuable clinical knowledge. All those reports and lectures have now been carefully revised—new matters added—and much expunged or condensed—so as to bring the whole within the compass of one volume—large indeed, and closely printed—but containing a library of practical medicine.

It is well known that the Dublin Schools, both medical and surgical, hold a very high rank among institutions of this kind, abroad and at home, and the professors have distinguished themselves, individually and collectively, in a very remarkable manner. The pages of this Journal bear ample testimony, for twenty years past, to the truth of this observation.

In the first introductory lecture of our author, he takes an opportunity of contrasting and comparing the modes of communicating clinical instruction in the hospitals of Edinburgh, London, Dublin, Paris, and Germany. They are all, more or less, deficient, but, upon the whole, he prefers the plan of our German brethren. As the surgical students concentrate all their attention on rare and terrible operations; so the medical students, in Great Britain especially, direct their chief observation to acute diseases, whose courses are well marked, and whose treatment is more simple than in chronic maladies. This applies, by the way, to the generality of young *practitioners*, as well as students. Yet, in their actual avocations, they will meet with fifty cases of chronic disease for one of acute. The Edinburgh CLINIQUE is fairly criticised. The clinical clerks take down the cases, which are read aloud at the bedside by the physician, and then the interrogatories, remarks, and treatment are vociferated loudly, that the crowd of students may hear, though they cannot see the case.

"The chief objection to this mode of teaching is, that however well inclined the student may be, he is never obliged to exercise his own judgment in distinguishing diseases, and has no opportunity of trying his skill in their cure; and, consequently, at the end of his studies he is perhaps well grounded in the accessory sciences—is a perfect medical logician—able to arrange the names of diseases in their classes, orders, and different subdivisions; he may be master of the most difficult theories of modern physiologists; he may have heard, seen, and, if a member of the Medical Society, he may have also talked a great deal; but at the end of all this preparation, what is he when he becomes a full Doctor? —a practitioner who has never practised!" 5.

In France, the mode of conveying clinical instruction, is very little different. There, however, no reports at the bed-side are dictated to the clerks, and more care is taken not to shock the feelings of the patients, by a full detail of the nature and consequences of their diseases. We now advert to the German Clinique.

"There is one clinical hospital for the treatment of acute diseases, and another for chronic diseases, while a clinical dispensary is devoted to the care of extern patients. The pupils are divided into two classes—the more advanced, who get the care of patients, and the junior students, who merely look on and listen. When a patient is admitted, his case is assigned to one of the practising pupils, who, when the physician is visiting the ward, reads out the notes he has taken of the patient's disease, including its origin, progress, and present state. This is done at the bedside of the patient; and before he leaves the ward, the physician satisfies himself whether all the necessary particulars have been accurately reported by the pupil. After all the patients have thus been accurately examined, the professor and his class proceed to the lecture-room, and a list of the patients and the practising pupils is handed to the professor: the cases admitted that day are first inquired into, and the pupils are examined concerning the nature of their diseases, their probable termination, and the most appropriate method of treatment,—each student answering only concerning the patients entrusted to his special care. During this examination, the pupil's diagnosis and proposed remedies are submitted to the consideration of the professor, who corrects whatever appears to be erroneous in either, and then the student retires to write his prescriptions, while the rest of the cases and pupils undergo a similar examination. At the conclusion, the prescriptions written by the students are read out in order by the professor, who strictly comments on and corrects any inaccuracy or inelegance they may contain. When the prescriptions have been revised and corrected, they are signed by the physician, and handed to the apothecary to be made up and distributed." 9.

Few will deny the propriety or utility of this plan; but few will adopt it—such is the inveteracy of habit, and the power of prejudice! Dr. Graves, however, conquered both, and introduced the German system, though with great difficulty. Dr. Graves entertains fears, and not without reason, that the modern attention to the collateral sciences—chemistry, electricity, magnetism, microscopic investigation, &c., will draw off much of the student's attention from the main object—CLINICAL OBSERVATION.

In the third introductory lecture Dr. Graves takes an opportunity of dissenting from some of the opinions of Liebig, (especially those respecting animal poisons and heat,) which are now "running the round of the journals," dazzling the optics of half the world—and almost eclipsing the "reflex-function" itself! In this attempt our author breaks a spear with Dr. Watson of the Middlesex.

It is not to be expected that we can give a review of a systematic work of this kind, occupying nearly a thousand pages:—we can only glance at some of the more prominent facts, doctrines, and opinions.

FEVER, as might be supposed, stands first on Dr. Graves's list, and is ably handled—Ireland being one of the most feverish countries of Europe, whether we view it in a moral, political, or pathological condition. Pyrexia is never absent from the Emerald Isle. Our author believes "typhus" to be a disease of "essential" character, and contagious. Dr. G. expects little or nothing from the chemical researches into the state of the blood in fever. He has long abandoned all attempts at forming a *theory* of the disease, and "confines himself altogether to a diligent study of its symptoms." He is wise. He has taught, for twenty years, "that morbid anatomy has not served to reveal the cause of fever." Our readers are aware that we have advocated the same in this Journal for 25 years past. The treatment of fever we must pass entirely over. As Dr. G. has no theory, he can have no specific modus medendi. He is an eclectic—watching phenomena, and combating them as well as he can. We may also pass over the post-mortem appearances in fever, as they have been described a thousand times by Continental and British physicians—and, after all lead but little to an elucidation of the nature of fever, or the best means of curing it. They are useful land-marks, however, to warn us of the rocks on which the life of the patient may perish, if the disease be not carefully watched.

In the 20th lecture, our author draws our attention to the causes of catarrhal affections of the bronchial tubes—especially chronic bronchitis—a disease more frequently met with in dispensary, as well as private practice, than almost any other. Nor is it very easily cured, for its causes are perpetually recurring, particularly among the lower classes of society. The part affected in bronchial inflammation produces great difference in the danger and treatment. In the larynx, for instance, it is of far more importance than in the trachea or bronchia. The following extract exhibits the practical tact of our author.

"You perceive, then, that if a patient catches cold, and gets an attack on the chest, it is of great importance to be able to ascertain what the situation and extent of the disease are, and whether the minute bronchial tubes are engaged or not. Now, how do you know this? Simply thus:—You first make a cursory examination of the whole chest, by applying the stethoscope over the superior, middle, and inferior portion of each lung, both before and behind; and, if you every where hear something, you conclude that the bronchitis is general, and not confined to any particular part. You next proceed to examine with greater attention these wheezing sounds; you apply the stethoscope, and if you find in each separate spot many sources of diseased sound—*if you hear a wheezing from a great many points close together*—you may be sure that the morbid sound proceeds from inflammation of the minute tubes, for the larger ones cannot exist in the small spots over which you apply the stethoscope, in such numbers as to give rise to so remarkable a plurality of sounds. Of this you may be certain, that when you find a great many sounds are audible over a small space, the minute bronchial ramifications are engaged.

It is the custom, with those who lecture on auscultation, to enumerate many sounds as connected with alterations in the condition of the bronchial tubes. We hear of the mucous, the sonorous, and the sibilant ronchus—their varieties and intermixtures. Now I know, by experience, that these names are very apt to confuse and perplex the young stethoscopist. There is no necessity for studying with great attention the definitions of these words, or the descriptions of the various sounds they are meant to represent: I am always anxious to avoid loading the memory of the student with names. With regard to the râles in bronchitis, all he need bear in mind is, that the nature of the sound produced by air passing through the bronchial tubes will be modified accordingly as these tubes are large or small, dry or moist, or as the moisture they contain is thin or not. The two things of greatest importance in examining a case of bronchitis is to ascertain whether the minute bronchial ramifications are engaged, and, if the tubes contain any moisture, whether it is thin or viscid.

"I seldom, therefore, confuse the student by telling him whether the râle is sibilant or sonorous, when asked about the nature of the sounds heard in a case of bronchial inflammation. All I say in reply is this: that the sounds are produced by the large or small bronchial tubes, and that they are either moist or dry." 228.

Dr. Graves gives a very able lecture on Hæmoptysis, its pathology and treatment. When a large quantity of blood is expectorated our author bleeds freely, and is not deterred by a depressed state of the vascular system. After venesection, he exhibits two grains of ipecacuanha every quarter of an hour, "until there is some improvement," and then every half-hour till the bleeding stops. He prefers this to acetate of lead; but does not reject the latter when combined with opium. Before any of these remedies are used, however, he prescribes acid saline aperients.

The 23rd lecture on Phthisis, notwithstanding the hacknied nature of the subject, will be perused with great advantage by many whose heads have grown grey in the practice of physic.

We must now make a long stride, in which "Gonorrhœa and Syphilis" occupies nearly one hundred pages, and all very ably handled. It is evident that Dr. Graves leans strongly to the non-mercurial treatment of syphilis; but he is not an "out-and-outer," as the following short quotation will testify.

"Notwithstanding all that has been said and done, a good deal still remains to be accomplished, before the treatment of syphilis can be said to be placed on a solid and rational basis. I am not among those who contend that you should never use mercury. On the contrary, I think there are cases in which you can employ it to great advantage—in fact, where its employment is indispensable. But I would have you always to act with caution. In treating cases of primary or secondary symptoms, which have existed for some time, and where the patient has been taking mercury, it is hard to unravel the perplexities which surround the case, and ascertain whether the mercury has been properly administered or not.

"Where a patient labouring under syphilis has been salivated without being improved, one of two things must be inferred—either that the mineral has had no effect on the disease, or that it has had an injurious effect on the constitution. The great point to arrive at in the treatment of syphilis is to make the mercury act on the disease, and not on the constitution. This I have often endeavoured to impress on my class. I will venture to say, that I would engage to give a patient labouring under primary symptoms any quantity of mercury, without producing a favourable effect on the disease, or doing him any good: I would engage to salivate a man affected with sore throat, and yet leave him as bad, or even worse, than ever. I have witnessed this occurrence over and over again, and have laid it down to myself as a proposition,—that venereal may be treated with mercury, to the fullest extent, without being cured." 369.

We shall be able to allude to only one more lecture, No. 30, on Paralysis. There is a great deal more of original thought, reflection, and observation in these lectures than in any systematic work which we have, for a long time, perused. Clinical lectures, from their semicolloquial character, and their constant reference to cases occurring in the wards of the hospital, are better adapted for the communication of original matter than the dry systems or ex professo treatises which the student and practitioner are doomed to wade through. Our author seldom plumes himself on this much prized quality of originality—except in the lecture now under consideration, where he considers his views as perfectly original, as far as he is acquainted. He does not treat of common paraplegia, occasioned by wounds, bruises, or diseases of the spinal column, marrow, brain, or membranes; but of paralysis occasioned by inflammation, irritation, or other disease or disorder of some remote part, as the stomach, bowels, kidneys—or even of the sentient extremities of the nerves of the skin, and where there is no organic lesion of either of the great nervous centres, or of the trunks of nerves leading to the affected members. Dr. G. explains this sympathetic paralysis by the *reflex*-function, using the term which Dr. Hall has long employed, but never alluding to that ingenious physician's theory. Dr. Graves descanted on this subject in his lectures delivered in 1832, and published in the "LONDON MEDICAL and SURGICAL JOURNAL" immediately afterwards. Mr. Stanley published his cases of sympathetic or functional paraplegia in the Transactions of the Medico-Chirurgical Society for 1833—so that the two authors may lay equal claim to *priority* (Hibernice) without damage to each other's originality. Both authors must have observed and collected their facts or data long before the æra of their publication. Besides, the journal in which Dr. Graves' lectures were published, had dwindled away, and was hardly read by any one at the time in question. The theory of our author is clearly and succinctly conveyed in the following sentence:—"It is, however, unnecessary to multiply examples to prove the truth of the proposition, that disease may commence in one portion of the nervous extremities, and be propagated towards the centre, and hence, by a reflex action, to other and distant parts." 398.

Dr. G. has collected, chiefly from his own experience, a very large mass of facts bearing on this obscure form of paralysis, which will amply repay the most careful perusal and re-perusal. We shall glance at a few of these.

*Case* 1. In Nov. 1832, our author attended a young gentleman, aged 14, who had eaten a large quantity of nuts, and suffered the usual penalties of pain, nausea, constipation, &c. These being removed by the usual means, enteric inflammation ensued, and was, with great difficulty, conquered. At length he was ordered to sit up, and then, to his astonishment, found that he was completely paraplegiac! Sensibility, however, remained, and the muscular power of the bladder and rectum was unaffected. It does not appear that this young gentleman recovered. Other cases are related where paralysis followed enteric inflammation.

"There can be little doubt that others have frequently noticed the occurrence of paraplegia after inflammation of the bowels, although no author has as yet written upon the subject. It is well to be acquainted with the occasional occurrence of so untoward and obstinate a sequela of enteric inflammation, in order that we may watch attentively the state of the lower extremities immediately after the inflammation of the bowels has been subdued." 404.

*Case* 2.—A sailor, aged 38, was admitted into the Richmond Hospital under Dr. Hutton, who had had several illnesses, but the last was occasioned by wet, cold, and fatigue, while on a passage from Cadiz to Dublin in 1834. He became affected with pain and weakness in his back and legs, with frequent micturition. He was cupped on the loins—diluents and opiates for the vesical irritation—bougies for a very close stricture in the membranous part of the urethra. "A very remarkable amendment took place in his back and lower extremities in a very few days after the introduction of the instrument," and he was soon discharged cured.

Cases are next related where paraplegia has followed fever, and without any apparent lesion of the spine or its contents. But we must conclude by stating the heads of a very remarkable case indeed.

*Case* 3.—Mr. B. aged 23, had been strong and hearty, passionately fond of hunting and shooting, and often getting wet in the bogs and moors. His bowels had been originally costive, but latterly relaxed. His "ATTACKS" were generally preceded by a copious discharge of insipid saliva from the mouth, followed by protracted nausea and vomiting of whatever happened to be in the stomach first, and afterwards of whatever he swallowed. The matter ejected was, in the beginning, acid and bitter, varying in colour, but generally green. The quantity thrown up in the day amounted to three or four quarts of fluid. There was pain in the stomach and lower part of the chest, of a constrictive nature. The first attack lasted four or five days, followed by an interval of seven months' good health, when the symptoms suddenly returned for a few days, and then went off. In 1830 he had three attacks, with intervals of health. In 1831, the paroxysms became much aggravated—lasted longer, and the intervals were shorter. For one of these attacks he took mercury to ptyalism. In 1832, all went on worse and worse, and he complained of numbness and some loss of power in the lower extremities, disappearing, however, when the vomiting subsided.

In August 1832, he had a violent attack, which lasted nearly a month—the vomiting continuing night and day incessantly. On getting up, after this attack, he found, to his astonishment, that he was paraplegiac. This paralysis did not disappear in the succeeding interval, though it was somewhat mitigated after the paroxysm, so that he could hobble along on two sticks. The next attack completed the paraplegia. His legs now wasted—lost sensation—and were cold to his own feelings. He had severe twitches of pain in various parts of the body.

"For some months before his death he was completely paraplegic, and continued to be attacked with violent fits of vomiting. The vomiting went on night and day, and he was unable to retain the mildest and most soothing substances for a moment on his stomach. Mr. Crampton and Dr. Ireland attended him with me, and we had recourse to every thing we could think of to allay the irritability of his stomach, but in vain. After continuing to resist obstinately every form of treatment for five or six days and nights, the vomiting would suddenly cease, the gentleman would exclaim, 'Now I am well,' and he could then eat with perfect impunity, substances which would prove irritating and indigestible to many stomachs. This was one of the most singular circumstances I ever witnessed. The transition from a state of deadly nausea and obstinate retching, to a sharp feeling of hunger used to occur quite suddenly. One hour he was the most miserable object you could behold, racked with painful constrictions across the epigastrium, alternately flushed or bathed with cold perspiration, and rejecting every thing from his stomach, the next found him eating with a voracious appetite whatever he could lay hold of, and digesting every thing with apparent facility."

"It may be observed that as the disease in this case proceeded, the intervals between the attacks diminished, while the paroxysms increased in duration. For the first two years they continued only for four or five days, and appeared at intervals of six or seven months; latterly they used to last for eight or ten days, and returned every third or fourth week. During the paroxysm the only thing which he took was a little cold water with some brandy and a few drops of laudanum, which remained longer on his stomach than any thing else, and enabled him to enjoy a few minutes sleep. He never complained of any headache, and his intellect was remarkably clear, and his memory good." 413.

He was worn out by this dreadful malady, and died on the 30th September, 1833. Every part of the body—head, spine, thorax, abdomen, &c. was most carefully examined, in the presence of excellent judges, and not an iota of change of structure could be detected any where!! Even the stomach, which bore the brunt of the terrible disorder for so long a time, "was perfectly healthy." Who, after this, will say that *functional disorder* will not destroy life, without leaving any trace of organic lesion? In respect to the point for which this case was introduced, it clearly proves that paraplegia may be produced by sympathy with some other or distant part—or by disordered function in the spinal marrow itself.

Here we must close our very imperfect notice of this valuable work, the nature and multifarious contents of which defy any thing like an analytical review, even if the whole of one of our numbers were dedicated to the attempt. We have little doubt, however, that these clinical lectures will obtain a very wide circulation, and a very attentive perusal.

